# SNHG1, a KLF4‐upregulated gene, promotes glioma cell survival and tumorigenesis under endoplasmic reticulum stress by upregulating BIRC3 expression

**DOI:** 10.1111/jcmm.17779

**Published:** 2023-05-26

**Authors:** Hongqiang Zhang, Binbin Ma, Na Li, Li Zhang, Jialu Xu, Shuqi Zhang, Ziming Guo, Chuanchun Han, Shasha Xu, Xiaodong Li, Bo Zhang

**Affiliations:** ^1^ Department of Neurosurgery, The Second Affiliated Hospital Dalian Medical University Dalian China; ^2^ Institute of Cancer Stem Cell, College of Basic Medical Science Dalian Medical University Dalian China; ^3^ Department of Gastroendoscopy the Fourth Affiliated Hospital of China Medical University Shenyang China; ^4^ Department of Neurosurgery, The Shenzhen Luohu Hospital Group The Third Affiliated Hospital of Shenzhen University Shenzhen China; ^5^ Neurosurgery Department of School of Medicine The Chinese University of Hong Kong Shenzhen China

**Keywords:** cell apoptosis, ER stress, glioma, SNHG1

## Abstract

Increasing evidence indicates that long noncoding RNAs (lncRNAs) play crucial roles in the resistance to endoplasmic reticulum (ER) stress in many cancers. However, ER stress‐regulated lncRNAs are still unknown in glioma. In the present study, we investigated the altered lncRNAs upon ER stress in glioma and found that small nucleolar RNA host gene 1 (SNHG1) was markedly increased in response to ER stress. Increased SNHG1 suppressed ER stress‐induced apoptosis and promoted tumorigenesis in vitro and in vivo. Further mechanistic studies indicated that SNHG1 elevated BIRC3 mRNA stability and enhanced BIRC3 expression. We also found that KLF4 transcriptionally upregulated SNHG1 expression and contributed to the ER stress‐induced SNHG1 increase. Collectively, the present findings indicated that SNHG1 is a KLF4‐regulated lncRNA that suppresses ER stress‐induced apoptosis and facilitates gliomagenesis by elevating BIRC3 expression.

## 1 BACKGROUND

Gliomas are the most common and malignant primary central nervous system tumours.^1^ Glioblastoma (GBM) accounts for 70% of all diffuse glioma diagnoses and is the most lethal brain tumour in adults. Although multiple advanced therapeutic strategies, including surgery, radiotherapy, chemotherapy and immunotherapy, have been used for the treatment of GBM, the 5‐year survival of patients is still less than 6%.^2^, ^3^ Thus, it is necessary to elucidate the molecular mechanism underlying the development and occurrence of gliomas.

While tumours quickly proliferate and spread, they usually are subjected to diverse hostile microenvironments, such as hypoxia, starvation and oxidative stress, which lead to endoplasmic reticulum (ER) stress.^4^, ^5^ Increasing evidence indicates that adaptation to ER stress and escaping from ER stress‐induced apoptosis have important impacts on cell survival, metastasis and therapeutic resistance in glioma.^6^, ^7^, ^8^ Therefore, understanding the mechanism of adaptation to ER stress is essential for developing new strategies for glioma treatment.

Small nucleolar RNA host gene 1 (SNHG1) is a long noncoding RNA which is located at chromosome 11q12.3 and is expressed in the nucleus of most cells as well as in the cytoplasm in some cell‐types.^9^ Accumulating evidence indicates that SNHG1 plays an oncogenic role in many cancers. SNHG1 promotes colorectal cancer cell growth by interacting with EZH2 and miR‐154‐5p,^10^, ^11^ and SNHG1 promotes colorectal cancer cell proliferation by regulating Wnt/β‐Catenin signalling.^12^ In breast cancer, SNHG1 enhances cell growth and metastasis by regulating macrophage M2‐like polarization.^13^ In hepatocellular carcinoma cells, SNHG1 contributes to sorafenib resistance by activating the AKT pathway and glycolysis to promote tumour progression.^14^, ^15^ In glioma, SNHG1 facilitates the malignant behaviours of glioma via the miR‐154‐5p/miR‐376b‐3p‐FOXP2‐KDM5B axis, and SNHG1 may also contribute to glioma progression by binding to miR‐194‐PHLDA1.^16^, ^17^ Although several reports have indicated the oncogenic role of SNHG1 in many cancers, the function and regulatory mechanism of SNHG1 under ER stress are still unknown.

In the present study, we found that SNHG1 was significantly upregulated under ER stress treatment. Increased SNHG1 suppressed ER stress‐induced glioma cell apoptosis and facilitated tumorigenesis. Further mechanistic studies indicated that SNHG1 increased BIRC3 mRNA stability and enhanced BIRC3 expression, which promoted glioma cell survival and tumorigenesis. Additionally, we also found that KLF4 transcriptionally upregulated SNHG1 expression and contributed to the ER stress‐induced SNHG1 increase. Collectively, the present findings indicated that SNHG1, a KLF4‐regulated lncRNA, suppresses ER stress‐induced apoptosis and facilitates gliomagenesis by upregulating BIRC3 expression.

## 2 MATERIALS AND METHODS

### 2.1 Cell culture and reagents

The U251 and T98G human glioma cell lines were maintained in Dulbecco's modified Eagle medium with 10% foetal bovine serum (FBS), 2 mM L‐glutamine, 100 U/mL penicillin and 100 U/mL streptomycin at 37°C in a humidified atmosphere with 5% CO_2_. The following antibodies were used in the present study: GAPDH (Santa Cruz Biotechnology; SC‐25778, 1:1000), PARP (Santa Cruz Biotechnology, SC‐8007, 1:1000), GRP78 (Santa Cruz Biotechnology, SC‐13968, 1:1000 for WB), KLF4 (Cell Signaling Technology, #12173S, 1:500) and BIRC3 (Proteintech, 24304‐1‐AP, 1:1000). SNHG1 siRNAs (horizon, R‐188051‐00‐0005), Tunicamycin (TM, Lot No. T7765) and thapsigargin (TG, Lot:T9033) was purchased from Sigma Chemical Co., and it was dissolved in DMSO in a stock solution of 3 mM for TM and 1 mM for TG. Cells were treated with 3 μM TM or 1 μM TG at the indicated times.

### 2.2 RNA‐sequencing analysis

U251 cells were treated with 3 μM TM for 36 h. Cells were then collected and sent to BioMaker where RNA extraction, library construction, sequencing and data analysis were performed.

### 2.3 Lentivirus packaging and infection

To generate lentiviral short hairpin RNA (shRNA) constructs against human SNHG1, KLF4 and BIRC3, the target sequences were cloned into the pLKO.1‐puro vector. The shRNA sequences are listed in Table S1. To generate lentivirus expressing SNHG1 and KLF4, a pCDH vector was constructed. To establish stable cell lines, the pLKO.1 vector, pVSVG, pREV and pGAG were cotransfected into HEK293T cells, or the pCDH vector, psPax2 and pMD2G were cotransfected into HEK293T cells. After 48 h, the viruses were collected and used to infect glioma cells as indicated. After 48 h, glioma cells were cultured in medium containing 2.5 mg/mL puromycin for the selection of stable clones. The knockdown or overexpression efficiency was evaluated by Western blot and qRT–PCR analyses.

### 2.4 Cell invasion assay

For cell invasion assays, control and treated cells in serum‐free medium were seeded into the upper well of a Transwell chamber (Millipore) precoated with Matrigel (BD Bioscience) and allowed to invade into the lower compartment containing medium supplemented with 10% FBS. The cells on the lower surface filter of each chamber were fixed with methanol, stained with 0.1% crystal violet and counted in five randomly selected microscopic fields.

### 2.5 Quantitative real‐time PCR (qRT–PCR)

The U251 and T98G glioma cell lines were treated with TM for the indicated times. RNA was isolated using TRIzol (Invitrogen), and 1 μg of total RNA was used to synthesize cDNA using the PrimeScriptTM RT reagent kit (Takara, RR047A) according to the manufacturer's instructions. The primers are listed in Table S1. The expression levels of these genes were normalized to those of β‐actin. Changes in gene expression were determined using the 2^−ΔΔCT^ method.

### 2.6 Cell viability and cell apoptosis assays

The viability of glioma cells treated with TM was determined by a CCK8 assay. In brief, U251 and T98G cells were seeded in 96‐well plates (5000 cells/well) and incubated overnight. Cells were then treated with TM as the control at the indicated concentrations for 36 h. The absorbance was measured with a spectrometer.

Cell apoptosis was detected using a cell apoptosis assay (YEASEN). Briefly, the indicated glioma cells treated with or without TM were harvested by centrifugation at 1000 × ***g*** for 5 min and then resuspended in 100 μL of binding buffer. Cells were then incubated for 10 min with 5 μL of Annexin V‐Alexa Fluor 488 and 10 μL of PI at room temperature in the dark. After the addition of 400 μL of binding buffer, cell apoptosis was detected by FACS (BD) analysis.

### 2.7 Promoter reporters and dual‐luciferase assay

The wild‐type and mutant promoters of SNHG1 were cloned into a pGL3‐basic vector. After transfection, luciferase activity was measured in a 1.5‐mL Eppendorf tube with a Promega Dual‐Luciferase Reporter Assay kit according to the manufacturer's protocol. Relative Renilla luciferase activity was normalized to firefly luciferase activity.

### 2.8 Chromatin immunoprecipitation (ChIP) assay

U251 cells were cross‐linked with 1% formaldehyde for 10 min at room temperature. A ChIP assay was performed using the anti‐KLF4 antibody and a ChIP kit (Millipore, Merck KGaA) according to the manufacturer's instructions. Anti‐rabbit IgG was used as the control. The bound DNA fragments were eluted and amplified by PCR, and the PCR products were separated by gel electrophoresis.

### 2.9 In vivo tumorigenesis assays

Animal research was performed according to the National Institute of Health Guide for the Care and Use of Laboratory Animals under the approval of the Animal Research Committee of Dalian Medical University. Male nude mice (4–6 weeks old and 18–20 g) were obtained from the SPF Laboratory Animal Center of Dalian Medical University (Dalian, China) and were randomly divided into the indicated groups. U251 cells with or without SNHG1 overexpression (5 × 10^6^ cells/100 μL) mixed with Matrigel (BD Biosciences) were injected subcutaneously into nude mice (*n* = 4/group). At Day 7 post injection of U251 cells, ER stress was induced in vivo by weekly intraperitoneal injections with 0.25 mg/kg TM for 4 weeks. For the control, a vehicle was intraperitoneally injected into the mice. Mice were sacrificed at the end of the experiment, and tumours were harvested and weighed.

### 2.10 Statistics and data analyses

Data are expressed as mean ± SD. Data were statistically evaluated using GraphPad Prism 5. Multiple comparisons between treatment groups and control groups were performed using Dunnett's least significant difference (LSD) test. *p* < 0.05 were considered statistically significant.

## 3 RESULTS

### 3.1 ER stress induces SNHG1 expression in glioma

**FIGURE 1:**
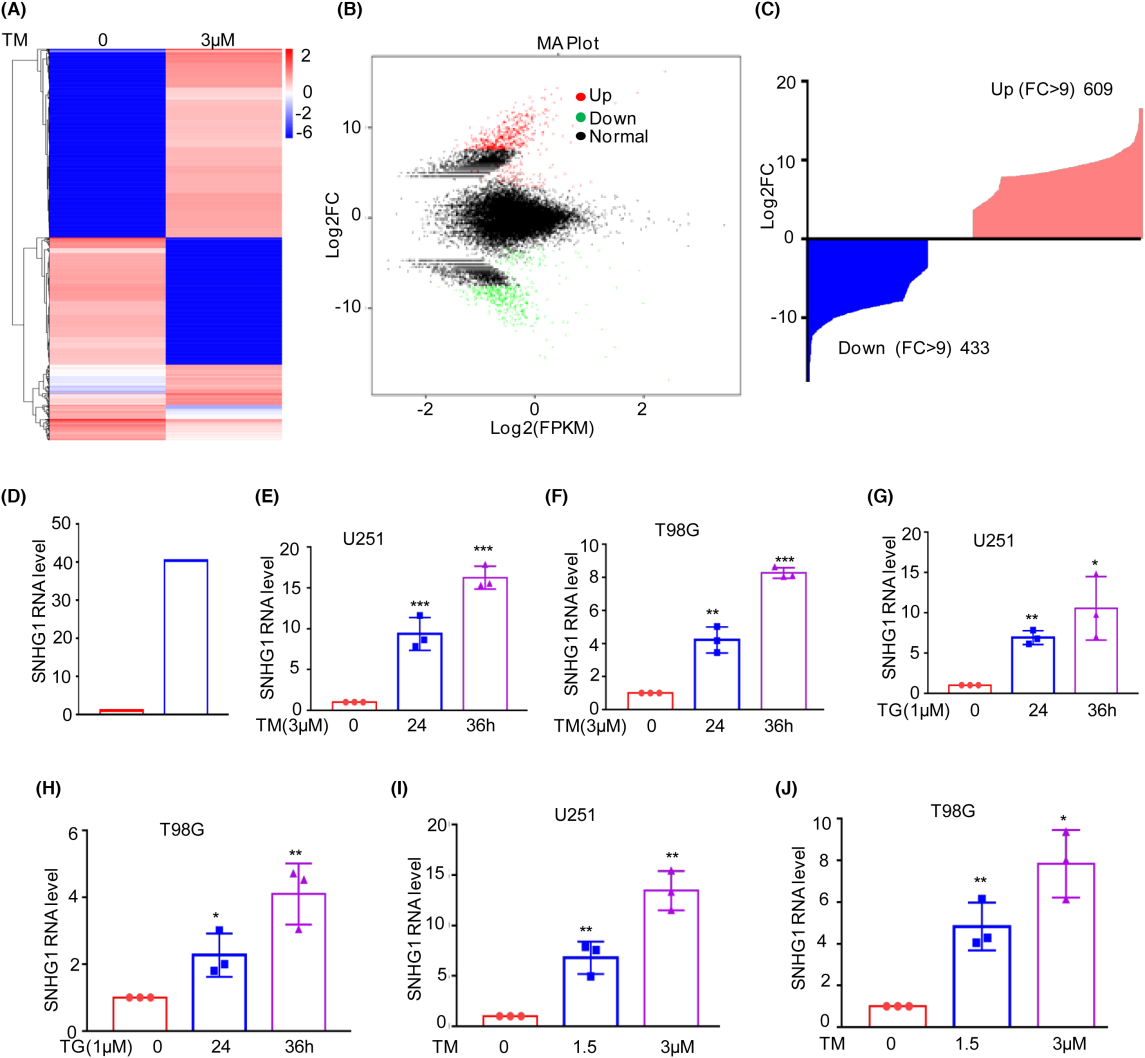
SNHG1 is increased under ER stress in glioma cells. (A–C) U251 cells were treated with or without 3 μM TM for 36 h and then subjected to RNA sequencing analysis. The altered lncRNAs are listed. (D) SNHG1 was selected for subsequent studies. (E–H) U251 and T98G cells were treated with 3 μM TM or 1 μM TG for the indicated times, and the expression levels of SNHG1 were measured by qRT–PCR. (I–J) U251 and T98G cells were treated with the indicated TM concentrations for 36 h, and the expression levels of SNHG1 were measured by qRT–PCR. Data in E, F, G, H, I and J were analysed by Student's *t*‐test (*p < 0.05, **p < 0.01 and ***p < 0.001).

To investigate the altered lncRNAs in response to ER stress, U251 glioma cells were treated with 3 μM TM to induce pharmacological ER stress, and cells were collected for RNA‐sequencing analysis. As shown in Figures 1A–C, 609 upregulated and 433 downregulated lncRNAs were obtained (Table S2). Among the changed lncRNAs, we found that SNHG1 was significantly increased in response to TM treatment (Figure 1D). To further confirm this hypothesis, U251 and T98G cells were treated with 3 μM TM or 1 μM thapsigargin (TG), a Ca^2+^‐ATPase inhibitor, to induce ER stress for the indicated times, and the expression levels of SNHG1 were analysed by qRT–PCR. Consistent with the RNA‐sequencing data, SNHG1 gradually increased with increasing ER stress treatment time (Figure 1E–H).

To determine whether the increase in SNHG1 is dependent on the concentration of TM, U251 and T98G cells were treated with the indicated doses of TM for 36 h, and the expression levels of SNHG1 were assessed by qRT–PCR. SNHG1 was upregulated with increasing doses of TM (Figure 1I,J). Taken together, these data suggested that SNHG1 is an ER stress‐induced lncRNA in glioma.

### 3.2 Knockdown of SNHG1 facilitates ER stress‐induced glioma cell apoptosis

**FIGURE 2:**
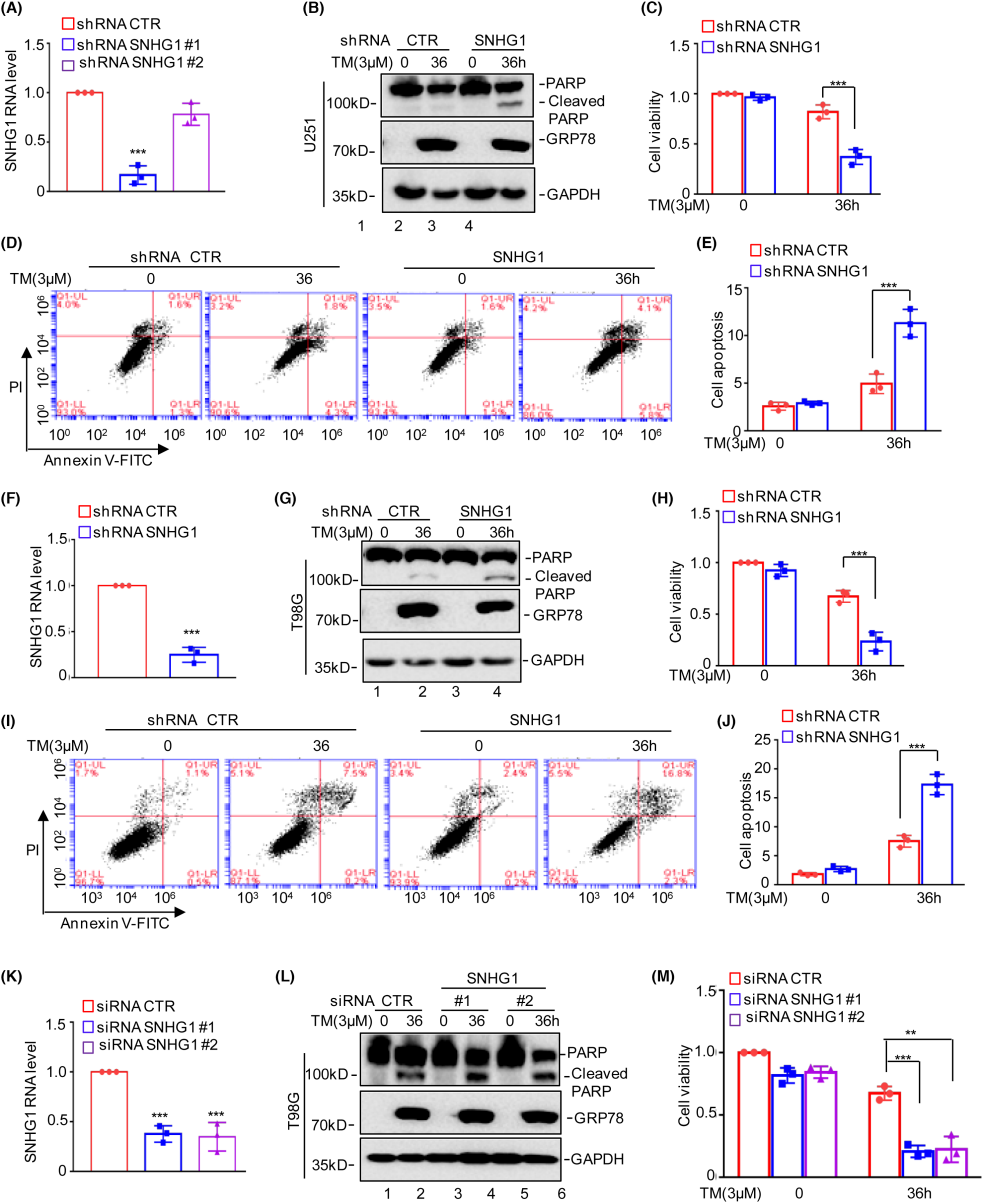
Knockdown of SNHG1 facilitates ER stress‐induced apoptosis and decreases cell viability in glioma cells. (A) SNHG1 was knocked down in U251 cells, and the knockdown efficiency of SNHG1 was measured by qRT–PCR. (B–E) Cell apoptosis and viability were analysed by Western blot analysis, CCK8 assays and flow cytometry assays. (F) SNHG1 was knocked down in T98G cells, and the knockdown efficiency of SNHG1 was measured by qRT–PCR. (G–J) Cell apoptosis and viability were analysed by Western blot analysis, CCK8 assays and flow cytometry assays. (K) SNHG1 was knocked down using two different siRNAs, and the expression of SNHG1 was measured by qRT–PCR. (L–M) Cell apoptosis and cell viability were analysed by Western blot analysis and CCK8 assays. Data in A, C, E, F, H, J, K and M were analysed by Student's *t*‐test (**p < 0.01 and ***p < 0.001).

To assess the biological role of SNHG1 in ER stress‐induced apoptosis, we first stably knocked down SNHG1 using lentivirus expressing shRNA in U251 cells, and the knockdown efficiency was measured by qRT–PCR. As shown in Figure 2A, the inhibition shRNA #1 was greater than that of shRNA #2. Thus, shRNA #1 was used in the subsequent studies. U251 cells with or without SNHG1 depletion were treated with 3 μM TM, and cell apoptosis and viability were detected. SNHG1 deficiency promoted ER stress‐induced apoptosis and a decrease in cell viability (Figure 2B–E). Similar results were obtained in T98G cells (Figure 2F–J). To exclude off‐target effects, siRNAs were used to knockdown SNHG1 in T98G cells, resulting in significant inhibition of SNHG1 expression (Figure 2K). Consistently, knockdown of SNHG1 enhanced ER stress‐induced apoptosis and promoted a decrease in cell viability (Figure 2L,M).

### 3.3 Overexpression of SNHG1 suppresses ER stress‐induced apoptosis and promotes gliomagenesis in vitro and in vivo

**FIGURE 3:**
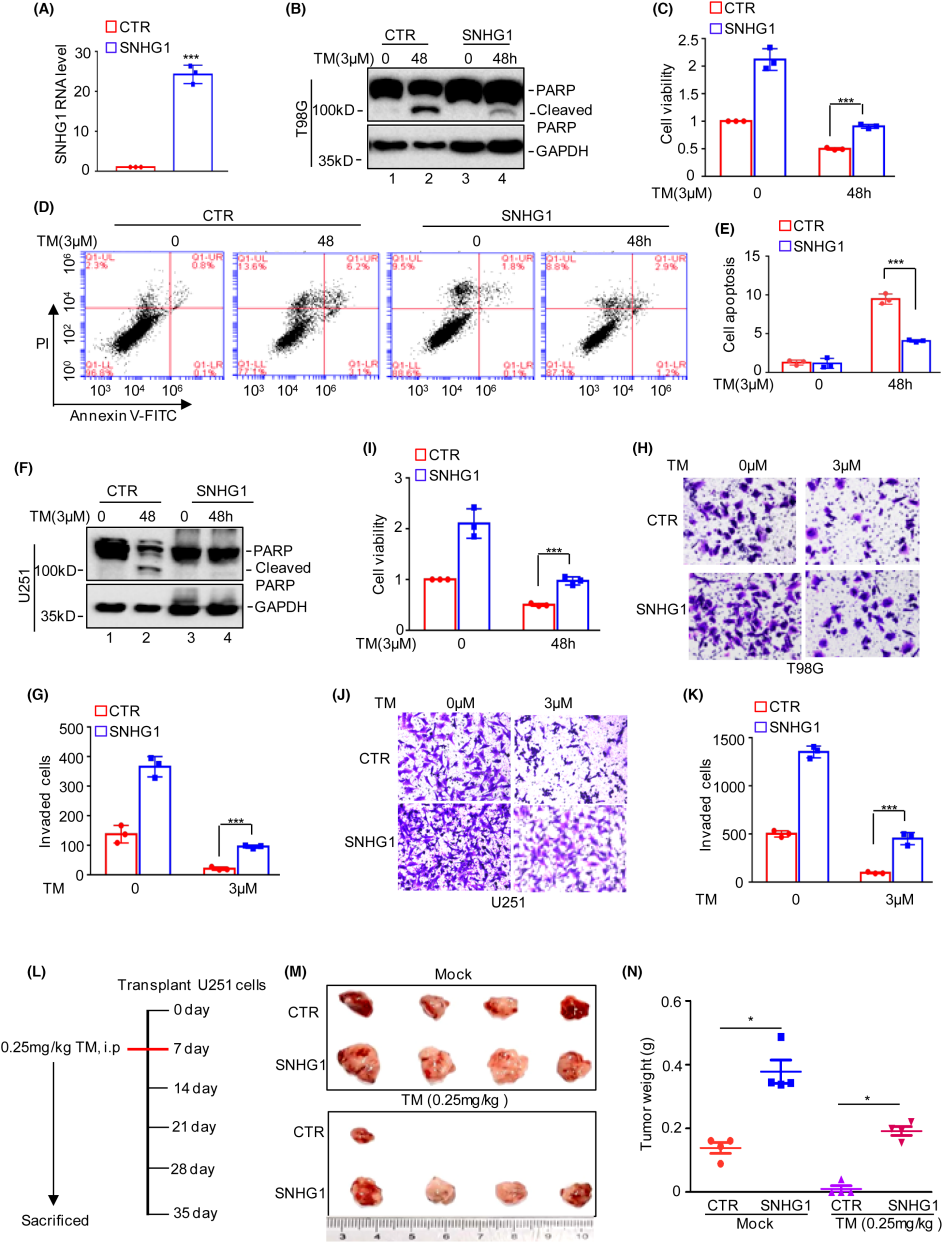
Overexpression of SNHG1 promotes glioma cell survival, invasion and tumorigenesis under ER stress. (A) SNHG1 was overexpressed in T98G cells, and the expression of SNHG1 was measured by qRT–PCR. (B–E) T98G cells with or without SNHG1 overexpression were treated with 3 μM TM for the indicated times. Cell apoptosis and viability were analysed by Western blot analysis, CCK8 assays and flow cytometry assays. (F–G) U251 cells with or without SNHG1 overexpression were treated with 3 μM TM for the indicated times. Cell apoptosis and cell viability were detected by Western blot analysis and CCK8 assays. (H‐K) T98G and U251 cells with or without SNHG1 overexpression were treated with or without 3 μM TM, and cell invasion was detected. (L) Scheme of treatment in the U251 xenograft tumour model with ongoing induced ER stress. (M‐N) The tumour images and tumour weights (dot plot) are presented. Data in A, C, E, G, I, K and N were analysed by Student's *t*‐test (*p < 0.05, and ***p < 0.001).

To further confirm the role of SNHG1 in ER stress‐induced apoptosis, SNHG1 was overexpressed in T98G cells (Figure 3A) followed by treatment with 3 μM TM for the indicated times, and cell apoptosis and viability were then assessed. As shown in Figures 3B–E, overexpression of SNHG1 decreased ER stress‐induced apoptosis and reversed ER stress‐induced cell viability downregulation. Similar results were obtained in U251 cells (Figure 3F,G). To better understand the role of SNHG1 under ER stress conditions, we investigated the effects of SNHG1 on cell invasion under ER stress. As shown in Figures 3H–K, elevated SNHG1 abolished the ER stress‐induced decrease in cell invasion in glioma cells. We then used xenograft models to assess the effect of SNHG1 on tumorigenesis under ER stress. U251 cells with or without SNHG1 overexpression were subcutaneously injected into nude mice. After 4 weeks of treatment with 0.25 mg/kg TM, overexpression of SNHG1 significantly promoted tumorigenesis and reversed the decrease in tumour size induced by TM treatment (Figure 3L–N). Collectively, these data indicated that SNHG1 plays an important role in ER stress‐induced apoptosis and facilitates gliomagenesis.

### 3.4 SNHG1 upregulates BIRC3 expression in glioma

**FIGURE 4:**
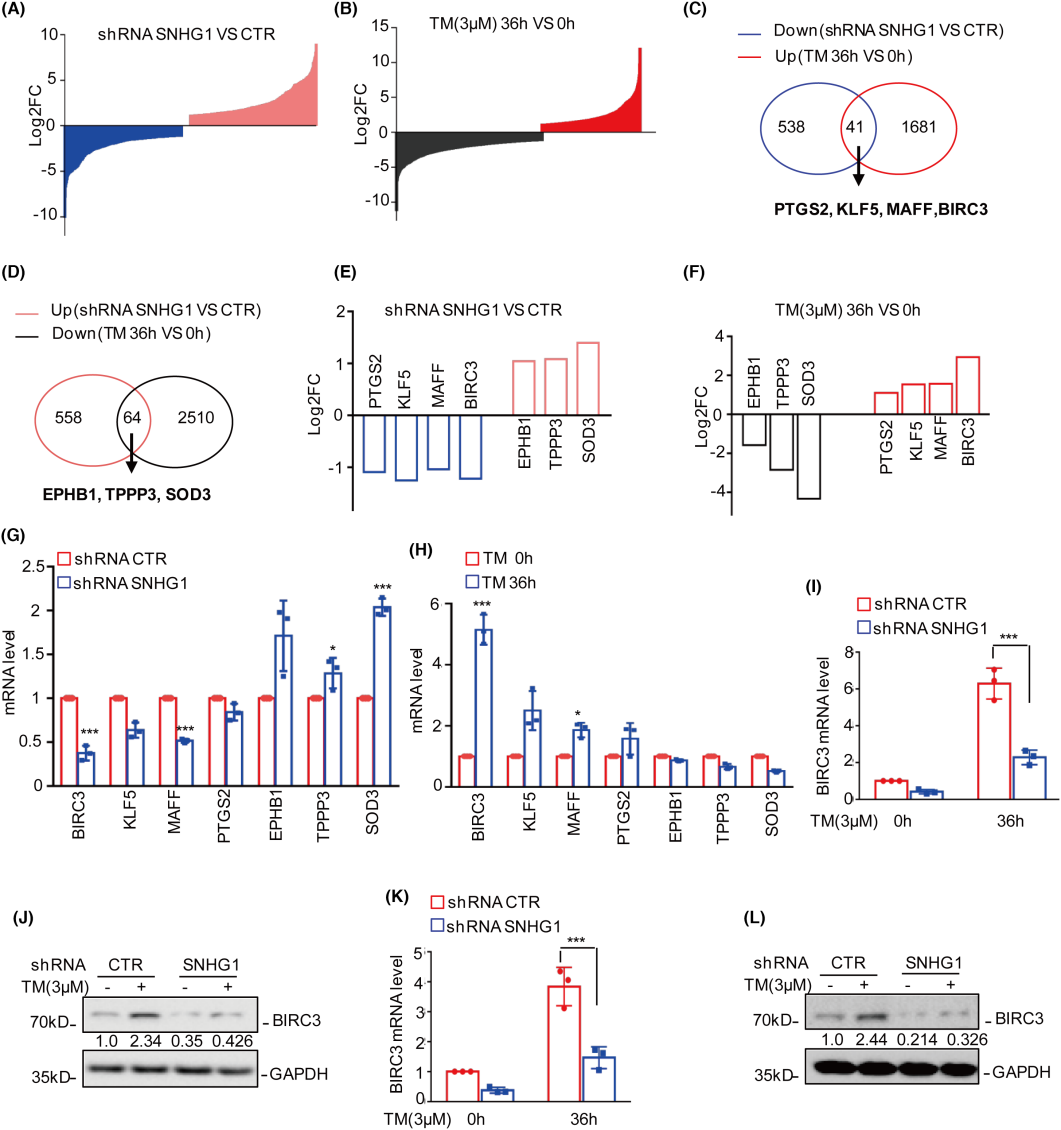
SNHG1 increases BIRC3 mRNA and protein levels. (A) U251 cells with or without SNHG1 knockdown were subjected to RNA sequencing analysis. The altered genes are listed. (B) U251 cells were treated with or without 3 μM TM for 36 h, and the altered genes were identified by RNA‐sequencing analysis. (C) Overlaps indicating the numbers of differentially expressed genes between the downregulated genes in SNHG1‐depleted cells and upregulated genes in TM‐treated cells. (D) Overlaps indicating the numbers of differentially expressed genes between the upregulated genes in SNHG1‐depleted cells and downregulated genes in TM‐treated cells. (E–F) The fold changes of selective genes are listed. (G) The altered genes were analysed by qRT–PCR in U251 cells with or without SNHG1 knockdown. (H) U251 cells were treated with or without 3 μM TM for 36 h, and the altered genes were analysed by qRT–PCR. (I–L) U251 and T98G cells with or without SNHG1 knockdown were treated with 3 μM TM for 36 h, and the expression levels of BIRC3 were evaluated by qRT–PCR and Western blot analysis. Numbers represent the relative intensities of Western blot bands of BIRC3 to GAPDH. Data in H, I, J, L were analysed by Student's *t*‐test (*p < 0.05, and ***p < 0.001).

To elucidate the molecular mechanism of SNHG1‐mediated inhibition of ER stress‐induced cell apoptosis, we used RNA‐sequencing analysis to identify the downstream genes of SNHG1 that are altered in response to ER stress. Compared to control cells, there were 558 upregulated and 538 downregulated genes in SNHG1‐deficient cells (Table S3). In addition, 1681 upregulated and 2510 downregulated genes were identified under ER stress(Table S4). Importantly, 41 downregulated and 64 upregulated genes had overlapping expression (Figure 4A–D). Based on the literature, we selected PTGS2, KLF5, MAFF, BIRC3, EPHB1, TPPP3 and SOD3 for further verification.^18^, ^19^, ^20^, ^21^, ^22^, ^23^, ^24^ The expression levels of these genes were evaluated by qRT–PCR analysis, and the fold changes are shown in Figures 4E,F. BIRC3 was significantly downregulated in SNHG1‐deficient cells (Figure 4H), and BIRC3 was significantly upregulated under ER stress, which was consistent with the SNHG1 expression (Figure 4I).

We next investigated whether BIRC3 is a downstream gene of SNHG1 in glioma. To this end, glioma cells with or without SNHG1 knockdown were treated with 3 μM TM, and the expression of BIRC3 was analysed by qRT–PCR and Western blot analyses. SNHG1 deficiency abolished the ER stress‐induced increase in BIRC3 expression (Figure 4J–M). Taken together, these data indicated that SNHG1 contributes to ER stress‐induced BIRC3 upregulation.

### 3.5 SNHG1 elevates BIRC3 mRNA stability and promotes glioma cell survival and invasion under ER stress conditions

**FIGURE 5:**
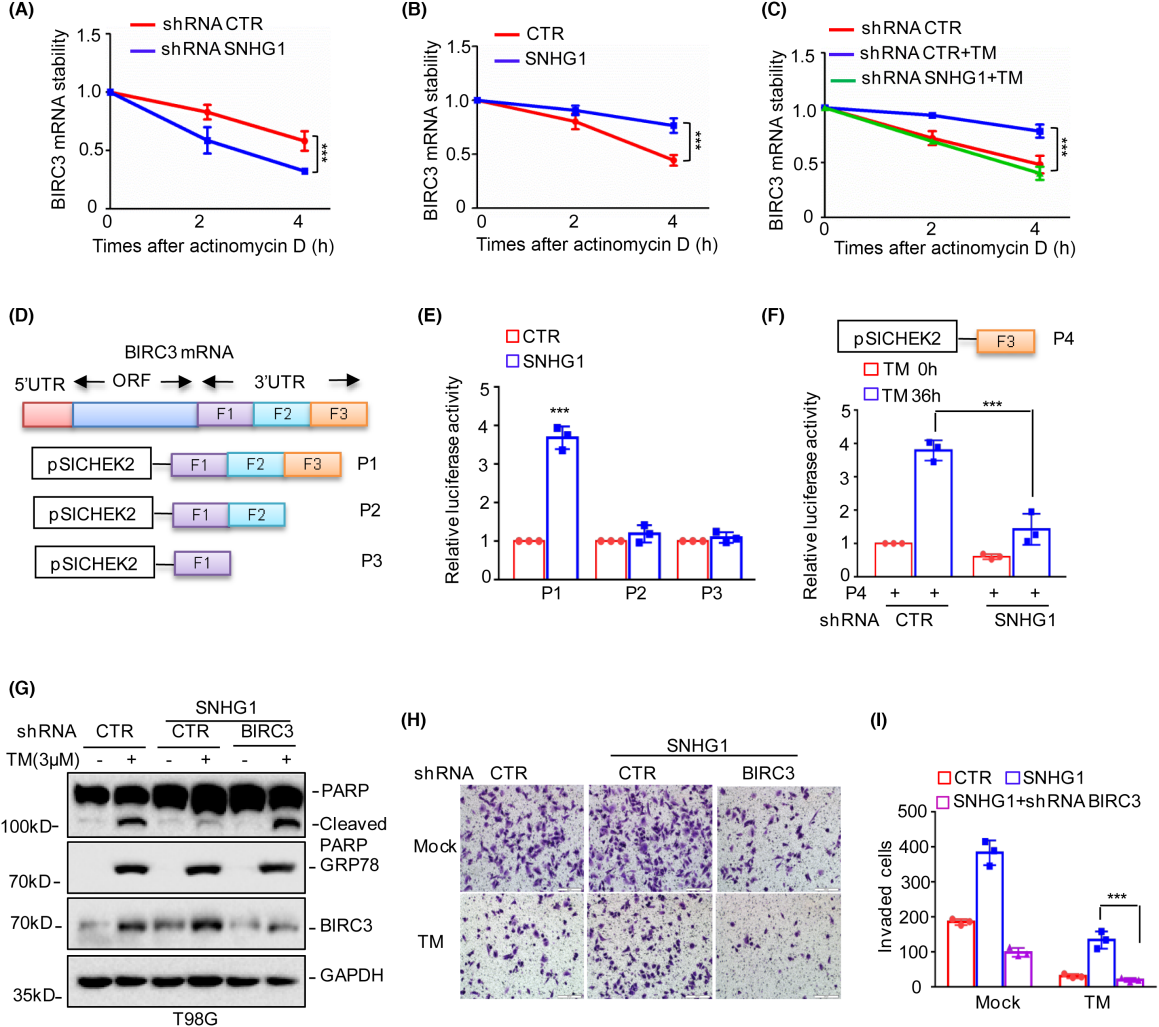
SNHG1 enhances the mRNA stability of BIRC3 and promotes glioma cell survival and invasion under ER stress. (A) U251 cells with or without SNHG1 knockdown were treated with actinomycin D (5 mg/mL) for the indicated times, and the mRNA stability of BIRC3 was measured by qRT–PCR. (B) T98G cells with or without SNHG1 overexpression were treated with actinomycin D (5 mg/mL) for the indicated times, and the mRNA stability of BIRC3 was measured by qRT–PCR. (C) U251 cells with or without SNHG1 knockdown were pretreated with 3 μM TM and then treated with actinomycin D (5 mg/mL) for the indicated times, and the mRNA stability of BIRC3 was measured by qRT–PCR. (D) Schematic of the transcript organization of BIRC3 mRNA and the 3′‐UTR of BIRC3 inserted into the pSICHECK2 plasmid. (E) P1, P2 and P3 were transfected into T98G cells with or without SNHG1 overexpression followed by measurement of luciferase activity. (F) The pSICHECK2 plasmid containing F3 of the BIRC3 3′‐UTR was transfected into U251 cells with or without SNHG1 knockdown followed by measurement of luciferase activity. (G) BIRC3 was knocked down in T98G cells with or without SNHG1 overexpression, and the cells were then treated with 3 μM TM. Cell apoptosis was detected by Western blot analysis. (H–I) Cell invasion was detected. Data in (A)–(C), (E), (F) and (I) were analysed by Student's *t*‐test (***p < 0.001).

To investigate the mechanism underlying the SNHG1‐mediated regulation of BIRC3 expression, we first examined whether SNHG1 directly regulates the transcription of BIRC3. To this end, we cloned the promoter of BIRC3 into the pGL3‐basic vector and then transfected the plasmid into T98G cells with or without SNHG1 overexpression, which demonstrated that elevated SNHG1 had no effect on the activity of the BIRC3 promoter (Figure S1). We next investigated whether SNHG1 affects BIRC3 mRNA stability by treating U251 cells with or without SNHG1 deficiency with actinomycin D to block de novo mRNA synthesis, and the mRNA stability of BIRC3 was measured by qRT–PCR. As shown in Figure 5A, SNHG1 deficiency decreased the mRNA stability of BIRC3 and promoted its mRNA degradation. In contrast, overexpression of SNHG1 elevated BIRC3 mRNA stability (Figure 5B). Moreover, ER stress elevated BIRC3 mRNA stability, but knockdown of SNHG1 abolished the ER stress‐induced increase in BIRC3 mRNA stability (Figure 5C).

Previous studies have indicated that the 3′‐UTR of BIRC3 plays an important role in regulating the stability of BIRC3 mRNA.^25^ To investigate whether SNHG1 upregulates BIRC3 mRNA stability through its 3′‐UTR, the 3′‐UTR of BIRC3 and different truncations were inserted into the pSICHECK2 vector, resulting in P1, P2 and P3 plasmids (Figure 5D). These plasmids were then transfected into T98G cells with or without SNHG1 overexpression, and firefly luciferase activity was measured. Dual luciferase reporter assays revealed that SNHG1 markedly elevated the luciferase activity of the 3′‐UTR of BIRC3, and the upregulation was abolished when the F3 fragment was depleted (Figure 5E). We also inserted the F3 fragment into the pSICHECK2 vector and measured the luciferase activity. TM significantly increased the luciferase activity of F3, and the increase was reversed when SNHG1 was knocked down (Figure 5F). Therefore, these data indicated that SNHG1‐mediated increases in BIRC3 mRNA stability rely on its 3′‐UTR and that the F3 fragment is essential for the upregulation of BIRC3 by SNHG1.

To further investigate whether SNHG1 suppresses ER stress‐induced apoptosis by upregulating BIRC3 expression, we knocked down BIRC3 in T98G cells with or without SNHG1 overexpression followed by treatment with 3 μM TM for the indicated times, and cell apoptosis was evaluated by Western blot analysis. As shown in Figure 5G, BIRC3 deficiency recovered ER stress‐induced apoptosis in SNHG1‐overexpressing glioma cells. Similar results were observed in the Transwell assays as knockdown of BIRC3 abolished the effect of SNHG1 on cell invasion under ER stress treatment (Figure 5H,I). Taken together, these data suggested that SNHG1 suppresses cell apoptosis and promotes cell metastasis by upregulating BIRC3 under ER stress conditions.

### 3.6 KLF4 transcriptionally upregulates SNHG1 expression

**FIGURE 6:**
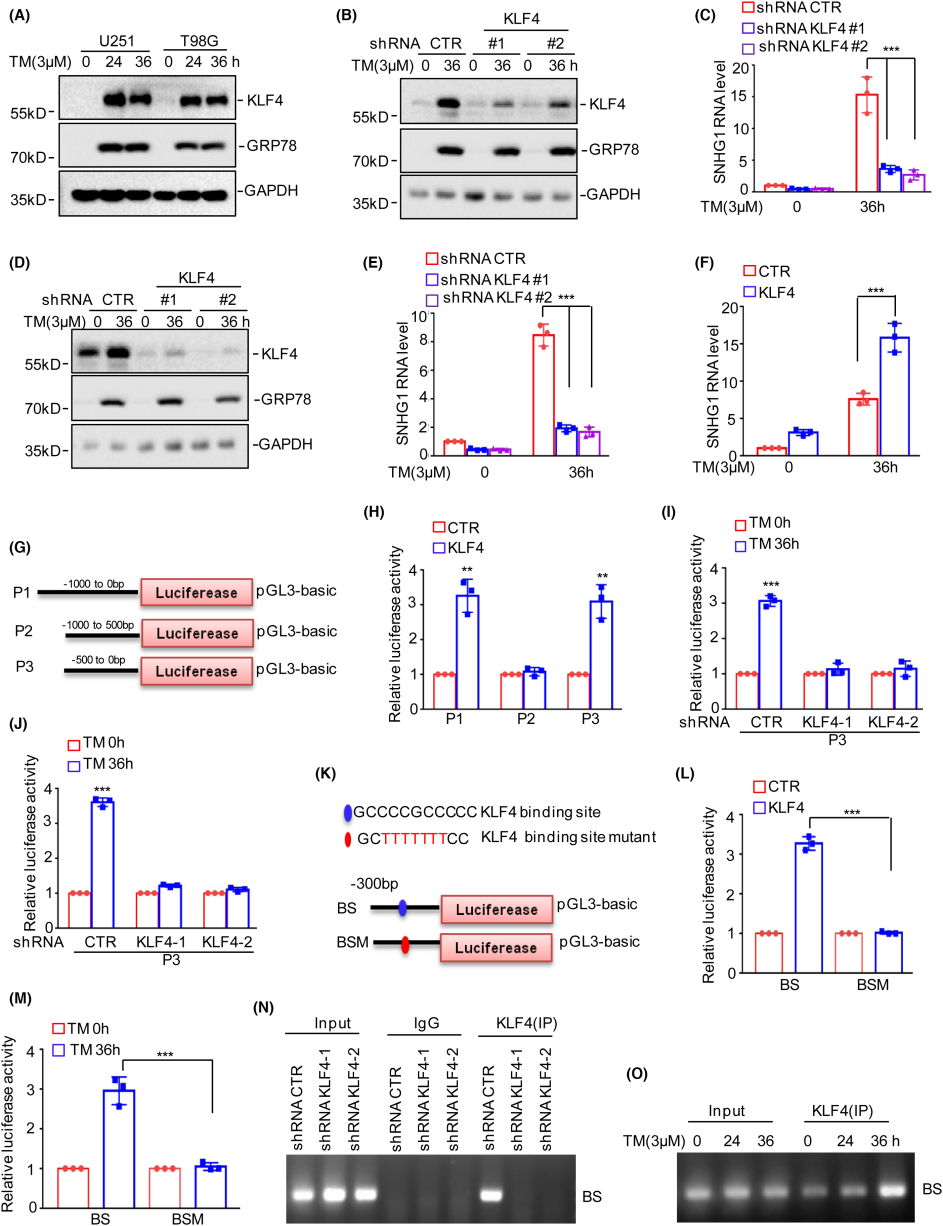
KLF4 transcriptionally upregulates SNHG1 under ER stress. (A) U251 and T98G cells were treated with 3 μM TM, and the expression levels of KLF4 were detected by Western blot analysis. (B–E) U251 and T98G cells with or without KLF4 knockdown were treated with 3 μM TM for the indicated times. The expression levels of SNHG1 were measured by qRT–PCR, and the protein levels of KLF4 were detected by Western blot analysis. (F) T98G cells with or without SNHG1 overexpression were treated with 3 μM TM for the indicated times, and the expression levels of SNHG1 were measured by qRT–PCR. (G) Schematic illustration of pGL3‐based reporter constructs used in luciferase assays to examine the transcriptional activity of SNHG1. (H) P1, P2 and P3 were transfected into 293T cells with or without KLF4 expression followed by measurement of luciferase activity. (I–J) P3 was transfected into U251 and T98G cells with or without KLF4 knockdown followed by measurement of the luciferase activity of P2. (K) Schematic illustration of the KLF4 wild‐type binding site (BS) and the matching mutant (BSM) used in the luciferase assays. (L) The wild‐type promoter (BS) or the matching mutant (BSM) was transfected into 293T cells with or without KLF4 overexpression followed by measurement of luciferase activity. (M) The wild‐type promoter (BS) or the matching mutant (BSM) was transfected into U251 cells with or without 3 μM TM treatment followed by measurement of luciferase activity. (M–O) ChIP analysis showing the binding of KLF4 to the promoter of SNHG1 in U251 cells with or without KLF4 knockdown or 3 μM TM treatment for the indicated times. Isotype‐matched IgG was used as a negative control. Data in (C), (E), (F), (H), (I), (J), (L) and (M) were analysed by Student's *t*‐test (**p < 0.01; ***p < 0.001).

Our previous studies indicated that KLF4 promotes gliomagenesis by upregulating ITGB4 expression and that KLF4 is an ER stress‐induced gene in melanoma.^26^, ^27^ Thus, we investigated whether KLF4 regulates SNHG1 expression in glioma. To this end, we first detected the expression of KLF4 in response to ER stress treatment and found that the protein levels of KLF4 were significantly increased upon TM treatment in glioma (Figure 6A). Subsequently, KLF4 was knocked down in U251 and T98G cells followed by treatment with 3 μM TM for 36 h, and the expression of KLF4 was measured by Western blot analysis (Figure 6B,D). As shown in Figure 6C,E, KLF4 deficiency abolished ER stress‐induced SNHG1 upregulation. In contrast, overexpression of KLF4 enhanced SNHG1 expression under ER stress in glioma (Figure 6F).

To further confirm that KLF4 transcriptionally upregulates SNHG1 expression, we cloned the upstream sequence of SNHG1 and different truncations by PCR and inserted them into the pGL3‐based luciferase reporter plasmid, and the plasmids were named P1–P3 (Figure 6G). Subsequently, we transfected the plasmids into 293 T cells with or without KLF4 overexpression. As shown in Figure 6H, KLF4 upregulated the luciferase activity of P1 and P3 but had no effect on the luciferase activity of P2, which indicated that the −500 to 0 bp region was essential for KLF4‐regulated SNHG1 expression. To confirm this hypothesis, U251 and T98G cells with or without KLF4 knockdown were transfected with P3 followed by treatment with 3 μM TM for the indicated times, and the luciferase activity of P3 was measured. The results indicated that knockdown of KLF4 abolished the ER stress‐induced increase in P3 luciferase activity (Figure 6I,J). To further identify the potential binding site of KLF4 on the promoter of SNHG1, we assessed the P3 sequence using the JASPAR database and found a positive KLF4‐binding site. To validate this hypothesis, two different pGL3‐based luciferase reporter plasmids containing the wild‐type (WT) and mutant (Mut) binding sites were constructed (Figure 6K). These plasmids were individually transfected into 293 T cells with or without KLF4 overexpression, and the luciferase activities of the WT and Mut BS were measured. The activity of WT but not Mut was significantly increased in response to KLF4 overexpression (Figure 6L). Similar results were obtained in U251 cells, in which TM significantly increased the luciferase activity of BS but not that of Mut (Figure 6M). These results indicated that BS is a positive KLF4‐binding site in the LINC00629 promoter.

We next performed a ChIP assay to demonstrate the specificity of chromatin fragments containing wild‐type BS in anti‐KLF4 immunoprecipitated samples from U251 cells with or without KLF4 knockdown or TM treatment. As shown in Figure 6N, the chromatin fragments (BS) were specifically present in the anti‐KLF4 immunoprecipitate. Moreover, the binding capacity of KLF4 to the SNHG1 promoter was impaired in KLF4‐deficient cells, and the binding capacity was enhanced upon TM treatment (Figure 6O). Collectively, these data suggested that KLF4 is a transcription factor of SNHG1 and contributes to SNHG1 upregulation under ER stress conditions.

### 3.7 KLF4 enhances BIRC3 expression and suppresses ER stress‐induced glioma cell apoptosis by upregulating SNHG1

**FIGURE 7:**
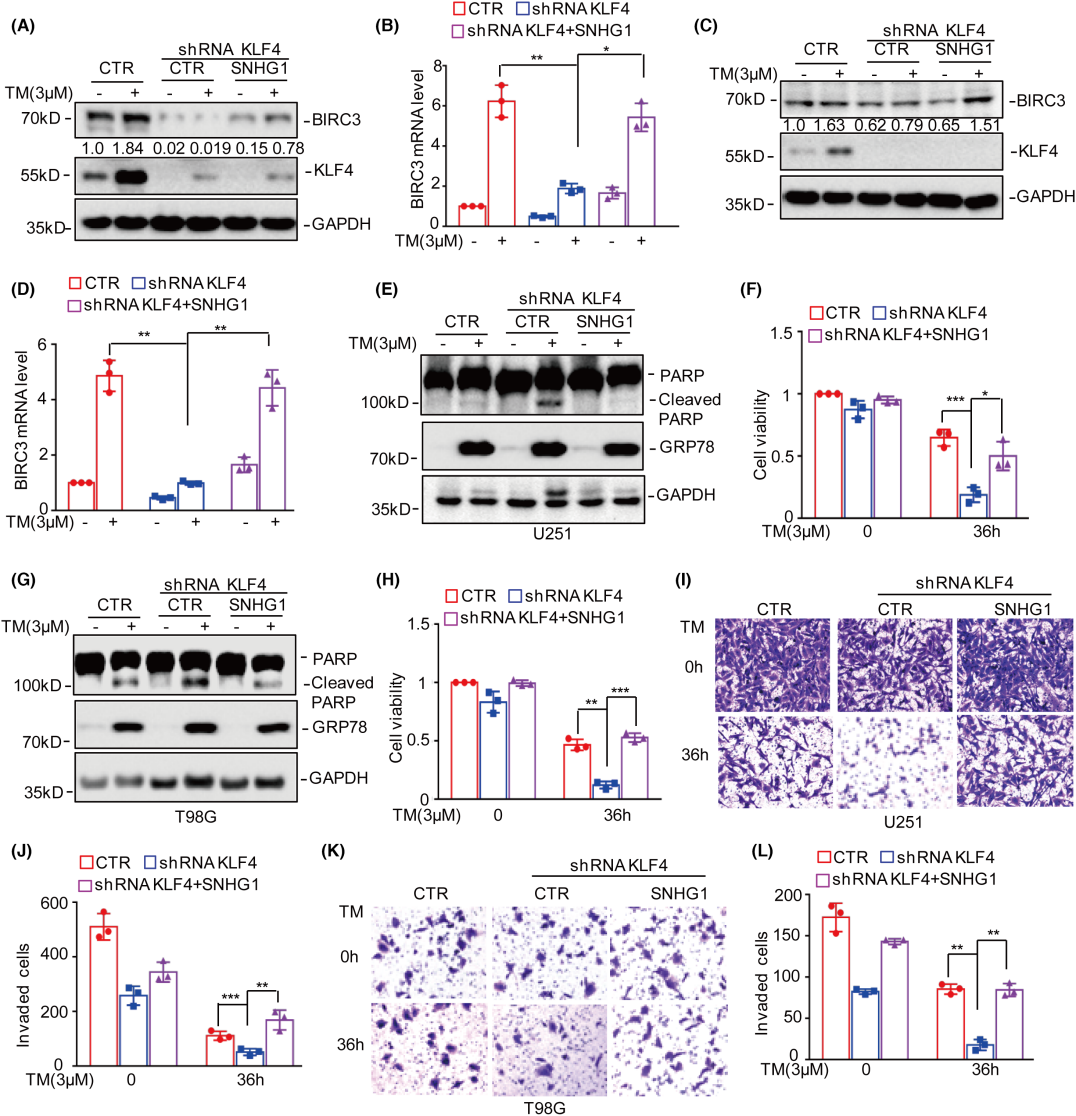
KLF4 promotes glioma cell survival, invasion and BIRC3 expression by regulating SNHG1 in response to ER stress treatment. (A–D) SNHG1 was overexpressed in U251 and T98G cells with or without KLF4 knockdown followed by treatment with 3 μM TM. The expression levels of BIRC3 were analysed by Western blot and qRT–PCR analysis. Numbers represent the relative intensities of Western blot bands of BIRC3 to GAPDH. (E–H) Cell apoptosis and viability were detected by Western blot analysis and CCK8 assays. (I–L) Cell invasion was detected by a Transwell assay. Data in (B), (D), (F), (H), (J) and (L) were analysed by Student's *t*‐test (*p < 0.05; **p < 0.01; ***p < 0.001).

Because SNHG1 upregulated BIRC3 expression under ER stress treatment, we next investigated whether KLF4 increases BIRC3 expression in a SNHG1‐dependent manner. SNHG1 was overexpressed in U251 and T98G cells with or without KLF4 knockdown followed by treatment with 3 μM TM for 36 h, and the expression of BIRC3 was assessed. As shown in Figure 7A–D, KLF4 deficiency impaired ER stress‐induced BIRC3 upregulation, which was then reversed by SNHG1 overexpression. Therefore, these data indicated that KLF4 upregulates BIRC3 expression in a manner dependent on SNHG1.

To determine whether KLF4 promotes glioma cell survival and invasion by regulating SNHG1, we overexpressed SNHG1 in glioma cells with or without KLF4 knockdown and then treated the cells with 3 μM TM. Cell apoptosis and viability were detected by Western blot analysis and CCK8 assays. The reduction in cell survival due to KLF4 deficiency was reversed when SNHG1 was overexpressed (Figure 7E–H). Similar results were observed in the Transwell assays, in which SNHG1 reversed the decrease in cell invasion induced by KLF4 knockdown. Taken together, these data indicated that KLF4 elevates BIRC3 expression and promotes cell survival and invasion by upregulating SNHG1 expression.

## 4 DISCUSSION

Glioma is a malignant tumour that rapidly proliferates and spreads. Moreover, gliomas are usually surrounded by harsh microenvironments, including hypoxia, starvation and oxidative stress, which lead to ER stress. However, the molecular mechanism by which glioma cells overcome hostile conditions and survive under ER stress is not completely understood. In the present study, we found that SNHG1, a KLF4‐upregulated gene, promoted glioma cell survival and invasion under ER stress by elevating BIRC3 expression.

SNHG1 is a noncoding RNA that hosts snoRNAs. Previous studies have indicated that SNHG1 is abnormally expressed in liver, prostate, colorectal, lung, gastric and oesophageal cancers, and increased SNHG1 expression contributes to cancer cell growth, metastasis and invasion.^12^, ^14^, ^28^, ^29^ In glioma, SNHG1 also plays an oncogenic role and facilitates the malignant behaviours of glioma cells. Upregulation of SNHG1 promotes cell proliferation and predicts poor prognosis. SNHG1 facilitates the malignant behaviours of glioma via the miR‐154‐5p/miR‐376b‐3p‐FOXP2‐KDM5B axis,^16^ and SNHG1 also contributes to glioma progression by binding to miR‐194‐PHLDA1.^17^ However, the role of SNHG1 in resistance to ER stress is still unknown. In our study, we demonstrated a novel biological role of SNHG1 in ER stress and found that SNHG1 was increased under ER stress treatment. Increased SNHG1 suppressed ER stress‐induced apoptosis and promoted cell metastasis.

To further elucidate the molecular mechanism by which SNHG1 inhibition promoted glioma survival and metastasis under ER stress, we used RNA sequencing to identify the downstream genes of SNHG1 and found that knockdown of SNHG1 downregulated the mRNA and protein levels of BIRC3.

BIRC3, also known as cellular IAP2 (cIAP2), is a member of the human IAP family.^30^ In glioma, high BIRC3 expression is associated with poor prognosis, and increased BIRC3 expression enhances glioma cell resistance to irradiation and temozolomide treatment. BIRC3 also promotes adaptation to hypoxic microenvironments in GBM.^22^, ^31^, ^32^ The present study indicated that SNHG1 enhanced BIRC3 mRNA stability and reduced its degradation in a manner dependent on the 3′‐UTR of BIRC3. However, it remains unknown how SNHG1 affects the activity of the 3′‐UTR of BIRC3, thereby warranting future studies.

Accumulating evidence indicates that some genes are involved in regulating SNHG1 expression. For example, METTL3 elevates the expression of SNHG1 by improving the RNA stability of SNHG1,^33^ and the SP1 transcription factor promotes SNHG1 expression and regulates bone remodelling and angiogenesis.^34^ In addition, E2F1 binds to the promoter of SNHG1 and upregulates SNHG1 in hepatocellular cancer.^15^ Similarly, we found that the KLF4 transcription factor also binds to the promoter of SNHG1 and contributes to ER stress‐induced SNHG1 upregulation in glioma.

## 5 CONCLUSIONS

**FIGURE 8:**
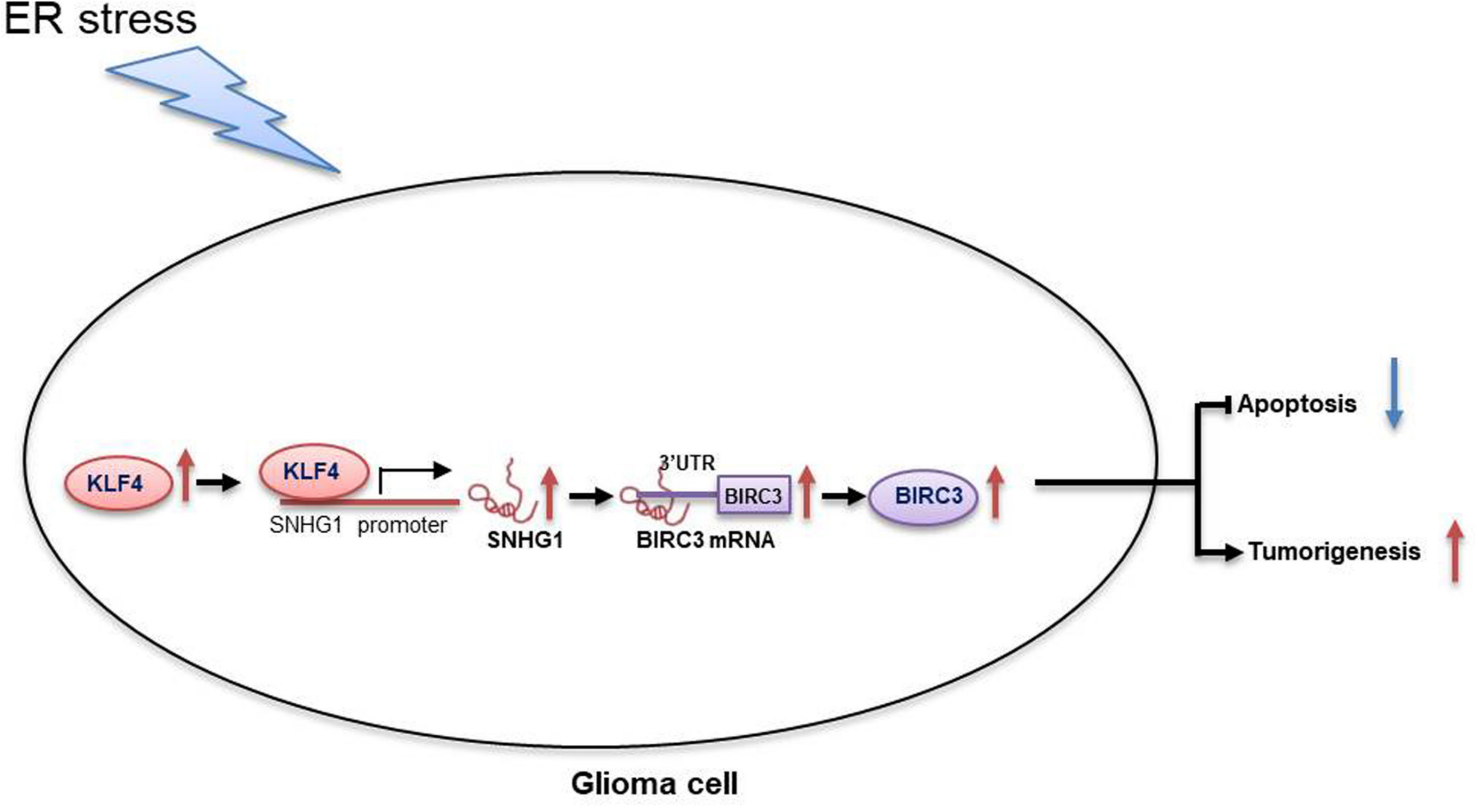
Schematic diagram of the mechanism by which SNHG1 promotes the adaptation to ER stress. SNHG1 is a KLF4‐regulated lncRNA that suppresses ER stress‐induced apoptosis and facilitates gliomagenesis by elevating BIRC3 expression.

In conclusion, the present findings indicated that SNHG1 is significantly increased in response to ER stress and that KLF4 transcriptionally upregulates SNHG1 expression, which suppresses ER stress‐induced apoptosis, thereby facilitating gliomagenesis by elevating BIRC3 expression (Figure 8).

## AUTHOR CONTRIBUTIONS

**Xiaodong Li:** Funding acquisition (equal); investigation (equal); writing – original draft (equal). **Hongqiang Zhang:** Data curation (equal); formal analysis (equal); investigation (equal). **Binbin Ma:** Formal analysis (equal); investigation (equal); writing – original draft (equal). **Na Li:** Formal analysis (equal). **Li Zhang:** Formal analysis (equal); investigation (equal). **Jialu Xu:** Resources (equal). **Ziming Guo:** Methodology (equal). **Chuanchun Han:** Investigation (equal); writing – original draft (equal). **Bo Zhang:** Investigation (equal); project administration (equal); supervision (equal). **Shuqi Zhang:** Formal analysis (equal). **Shasha Xu:** Methodology (equal); writing – review and editing (equal).

## FUNDING INFORMATION

This research was supported by the National Natural Science Foundation of China (No. 81902541 to Binbin Ma), the Basic Scientific Research Project by Department of Education in Liaoning Province 2021 (General Project, No. LJKZ0837 to Xiaodong Li) and Liaoning Provincial Natural Science Foundation of China (No. 2023‐MS‐264).

## CONFLICT OF INTEREST STATEMENT

The authors declare no competing interests.

## Supporting information


Figure S1.

Table S1.

Table S2.

Table S3.

Table S4.

## Data Availability

The data that supports the findings of this study are available in the manuscript and supplementary materials.
