# Supplementary figures and images for: SNHG1, a KLF4‐upregulated gene, promotes glioma cell survival and tumorigenesis under endoplasmic reticulum stress by upregulating BIRC3 expression
Source: J Cell Mol Med. 2023 May 26;27(13):1806–19. doi: 10.1111/jcmm.17779 (PMC10315853; doi:10.1111/jcmm.17779)

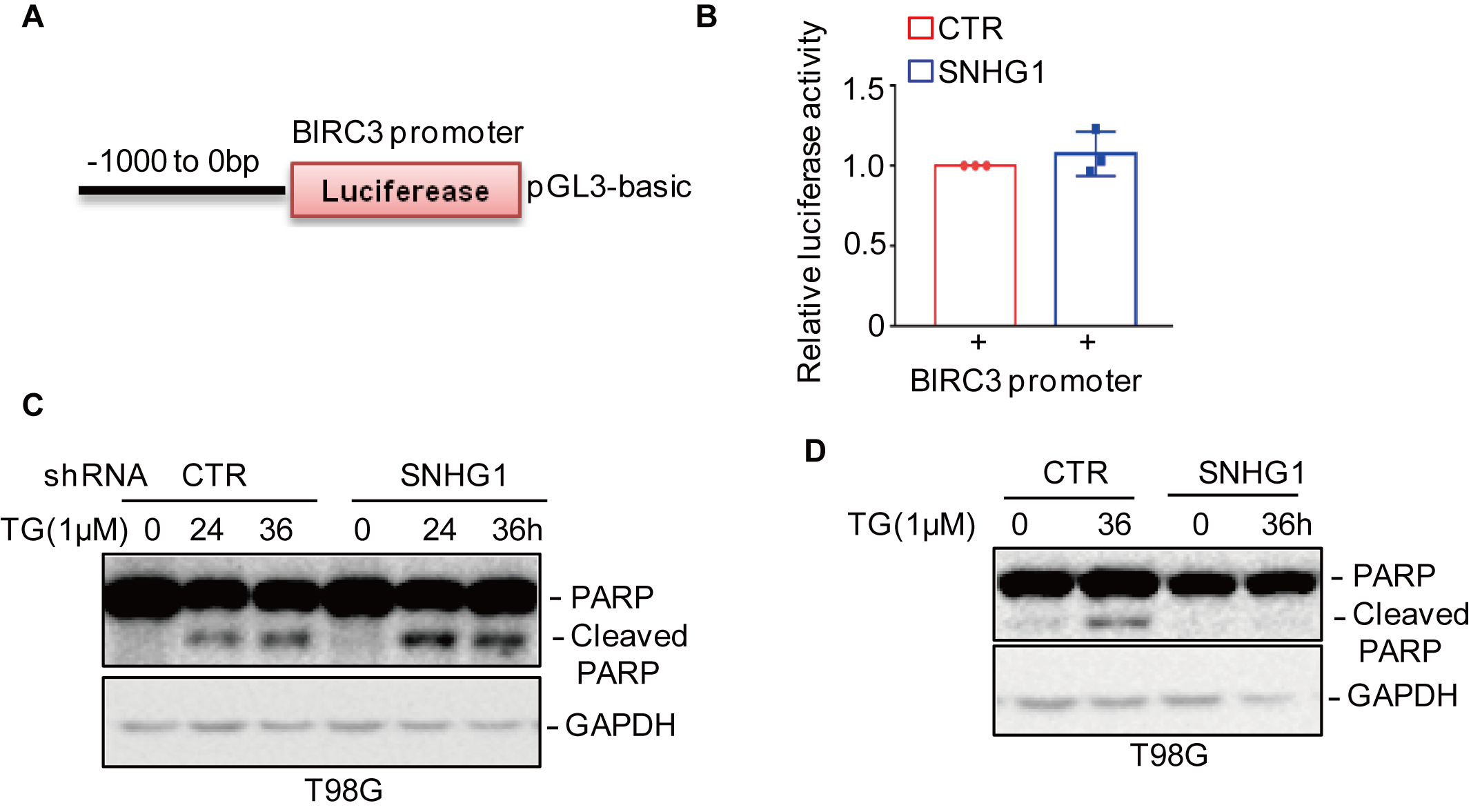

Supplement: Supplementary file 1 — Figure S1. [file JCMM-27-1806-s001.tif]
